# Next-level vegetation health index forecasting: A ConvLSTM study using MODIS Time Series

**DOI:** 10.1007/s11356-024-32430-x

**Published:** 2024-02-14

**Authors:** Serkan KARTAL, Muzaffer Can IBAN, Aliihsan SEKERTEKIN

**Affiliations:** 1 Department of Computer Engineering, Çukurova University, 01380 Adana, Türkiye; 2 Department of Geomatics Engineering, Mersin University, Yenişehir, 33110 Mersin, Türkiye; 3 Vocational School of Higher Education for Technical Sciences, Department of Architecture and Town Planning, Igdir University, 76002 Igdir, Türkiye

**Keywords:** Remote sensing, Forecasting, MODIS, ConvLSTM, Vegetation Health Index (VHI), NDVI, LST

## Abstract

The Vegetation Health Index (VHI) is a metric used to assess the health and condition of vegetation, based on satellite-derived data. It offers a comprehensive indicator of stress or vigor, commonly used in agriculture, ecology, and environmental monitoring for forecasting changes in vegetation health. Despite its advantages, there are few studies on forecasting VHI as a future projection, particularly using up-to-date and effective machine learning methods. Hence, the primary objective of this study is to forecast VHI values by utilizing remotely sensed images. To achieve this objective, the study proposes employing a combined Convolutional Neural Network (CNN) and a specific type of Recurrent Neural Network (RNN) called Long Short-Term Memory (LSTM), known as ConvLSTM. The VHI time series images are calculated based on the Normalized Difference Vegetation Index (NDVI) and Land Surface Temperature (LST) data obtained from the Moderate Resolution Imaging Spectroradiometer (MODIS) aboard the Terra and Aqua satellites. In addition to the traditional image-based calculation, the study suggests using global minimum and global maximum values (global scale) of NDVI and LST time series for calculating the VHI. The results of the study showed that the ConvLSTM with a 1-layer structure generally provided better forecasts than 2-layer and 3-layer structures. The average Root Mean Square Error (RMSE) values for the 1-step, 2-step, and 3-step ahead VHI forecasts were 0.025, 0.026, and 0.026, respectively, with each step representing an 8-day forecast horizon. Moreover, the proposed global scale model using the applied ConvLSTM structures outperformed the traditional VHI calculation method.

## Introduction

Since the inception of agriculture, the relationship between weather patterns and crop yield has consistently been a source of concern. The potential impact of yield variability on food security has been a driving force behind this ongoing concern. Although advancements in technology, including innovations such as irrigation, new seed varieties, fertilizers, greenhouses, and land management techniques, have significantly alleviated the direct influence of weather on agriculture, weather-related shocks continue to pose a substantial threat to stakeholders across the agricultural value chain (Hammad and Falchetta 2022). Beyond the realm of immediate weather variability, numerous studies underscore the considerable and predominantly negative impact of anthropogenic climate change on crop growth and overall health. The anticipated outcome is detrimental to global crop yields, ultimately posing challenges to food supply and accessibility (Abbass et al. 2022; Lyon et al. 2022). The IPCC (2021) predicts that a 0.5 °C increase in the global average temperature will likely lead to more frequent and severe weather-related vegetation health problems. This impact is particularly pronounced in the Mediterranean region due to significant variations in precipitation and prolonged periods of low rainfall. Additionally, large areas in this region are dedicated to agriculture, which puts a high demand on water resources (Bento et al. 2018). Given this evolving landscape and the pessimistic outlook, the imperative has emerged to monitor and accurately predict vegetation health.

Recent years have witnessed increasing use of remote sensing data for vegetation health monitoring, with a focus on indicators such as vegetation index or surface temperature. These indicators have different roles in monitoring vegetation health (West et al. 2019). The Vegetation Health Index (VHI) is a well-known index based on remote sensing data, which is a combination of the Vegetation Condition Index (VCI) and the Thermal Condition Index (TCI). The Normalized Difference Vegetation Index (NDVI) is used to estimate the VCI to determine vegetation water stress by combining information from the visible and near-infrared portions of the electromagnetic spectrum. On the other hand, the TCI is calculated using Land Surface Temperature (LST) and is utilized to evaluate vegetation temperature stress (Bento et al. 2020). Many researchers have used these three vegetation-related indices to study agricultural productivity and track vegetation behavior in response to weather-related shocks.

The motivation behind this study stems from the critical need to advance the forecasting capabilities of the VHI. Existing literature predominantly focuses on time series forecasting models for various remote sensing indices, such as NDVI and LST. Still, there is a noticeable gap in forecasting the VHI, which integrates both vegetation conditions and temperature stress. Previous methods for forecasting remote sensing indices have exhibited certain limitations that this study seeks to overcome. While effective for stationary time series, traditional time series forecasting models face challenges in handling the complex non-linear dependencies inherent in large, multi-variable datasets. Additionally, traditional machine learning methods may struggle with the intricate spatiotemporal variability and high-dimensional inputs present in remote sensing datasets. The study aims to fill this gap by introducing a novel approach that employs Convolutional Long Short-Term Memory (ConvLSTM) networks for forecasting VHI values. To do so, we used publicly available remote sensing data from the Moderate Resolution Imaging Spectroradiometer (MODIS) satellite sensor, commonly considered for earth and climatic observations.

This methodology represents a significant advancement, as it not only addresses the limitations of traditional time series forecasting models but also extends the application of ConvLSTM to the unique challenges posed by the VHI. The use of three different ConvLSTM structures and testing various forecasting intervals adds depth to the analysis, allowing for a comprehensive evaluation of predictive accuracy over different time horizons. Furthermore, the study introduces a novel "global scale" approach, incorporating global minimum and maximum values, which enhances the robustness of the forecasting model.

The potential contributions of this research are substantial, providing a pioneering exploration into forecasting the VHI. First, by leveraging the capabilities of the ConvLSTM networks, the study surpasses the limitations of traditional time series forecasting models. The application of the ConvLSTM networks has demonstrated superior performance in forecasting time series data for NDVI and LST (Gavahi et al. 2021; Ahmad et al. 2020; Kartal and Sekertekin 2022). To the best of our knowledge, this study represents the pioneering effort to implement the ConvLSTM networks specifically for forecasting the VHI time series. By introducing the ConvLSTM to the forecasting framework for VHI, this research aims to elevate the precision and effectiveness of VHI forecasts, marking a significant advancement in vegetation health monitoring.

Second, the findings have implications for resource management, agriculture, and environmental monitoring, offering valuable insights for decision-makers and stakeholders in these domains. In agriculture, the ability to forecast the VHI through advanced deep learning techniques contributes significantly to crop yield prediction and management. By understanding the anticipated health of vegetation in advance, farmers and agricultural stakeholders can strategically arrange planting times, irrigation schedules, and crop rotations. This awareness not only enhances agricultural productivity but also minimizes the impact of potential drought conditions, fostering more resilient and sustainable farming practices. Moreover, in the context of environmental monitoring, the study’s findings have broader implications for assessing the overall health of ecosystems and biodiversity. Accurate VHI forecasting aids in the early detection of potential stress on vegetation, offering a proactive approach to addressing environmental challenges. Besides, it is particularly crucial for preserving ecosystems, protecting wildlife habitats, and mitigating the impact of climate change on diverse plant species.

### Review of using remote sensing data for vegetation health monitoring

Monitoring vegetation health using remote sensing data allows for the assessment of the condition or health of the vegetation cover in response to changing weather conditions (precipitation and/or temperatures). Remote sensing products detect the electromagnetic energy emitted by objects. In theory, healthy vegetation should emit more in the near-infrared (NIR) portion of the electromagnetic spectrum than in the visible red (RED). As vegetation loses health, it reflects more RED and less NIR radiance. This theory led to the development of the NDVI, which is calculated by dividing the difference between the RED and NIR radiance by the sum (Kriegler et al. 1969), as shown in Eq. (1).

1 $$NDVI= \frac{\left(NIR-RED\right)}{\left(NIR+RED\right)}$$

Kogan (1990) recommends using the VCI to determine whether there is a drought. VCI helps monitor agricultural droughts and gives extensive data during the farming season. It serves as a gauge of the state of the vegetation cover by utilizing the lowest and highest NDVI values gathered in a specific time interval. It scales between the maximum and minimum NDVI values for normalization. The VCI is calculated using NDVI values, as shown in Eq. (2).

2 $$VCI=\left(\frac{NDVI-{NDVI}_{min}}{{NDVI}_{max}-{NDVI}_{min}}\right)$$

where $VCI$ is the vegetation condition index at the time of observation, $NDVI$ is the NDVI value at the time of observation. While ${NDVI}_{min}$, and ${NDVI}_{max}$ correspond to the minimum and maximum NDVI values in an image within the image-based dataset, respectively. They correspond to the minimum and maximum NDVI values of each pixel (location) in the temporal dimension in the global scale dataset. However, a VCI model based merely on NDVI is insufficient for monitoring vegetation health, and a temperature-related indicator is also necessary to understand the thermal stress on vegetation cover.

Therefore, the TCI is used to measure this temperature stress on vegetation cover. During dry seasons, soil moisture levels decline significantly, putting the plants under heat stress. Thus, higher temperatures cause dryness throughout the vegetation growth cycle, whereas low temperatures stimulate vegetation growth. The TCI is computed using the LST values (Kogan 1995), as shown in Eq. (3).

3 $$TCI=\left(\frac{{LST}_{max}-LST}{{LST}_{max}-{LST}_{min}}\right)$$

where $LST$ corresponds to the LST value at the time of observation. While ${LST}_{min}$ and ${LST}_{max}$ correspond to the minimum and maximum LST values in an image within the image-based dataset, respectively. They correspond to the minimum and maximum LST values of each pixel (location) in the temporal dimension in the global scale dataset. Low TCI levels imply extremely hot weather, which may result in drought depending on its severity.

In the end, Kogan (2002) developed the VHI, which is a weighted average of the VCI and TCI, to integrate vegetation conditions and temperature stress. The VHI measures the health of vegetation cover by linking stressed conditions with low NDVI and high temperatures. The VHI is calculated using TCI and VCI values, as shown in Eq. 4.

4 $$VHI= \alpha *VCI+\left(1-\alpha \right)*TCI$$

Since the appropriate weights of the VCI and TCI components are generally unknown, the VHI calculation assigns an alpha weight of 0.5 to each (Yagci 2021).

All of these indices range from 0 to 1. VCI values greater than 0.50 indicate a healthy condition for vegetation cover, while values less than 0.10 indicate very unhealthy conditions. Similarly, TCI values exceeding 0.40 indicate normal thermal conditions for vegetation cover, while values less than 0.10 mean the vegetation cover is under severe thermal stress. Finally, VHI values more than 0.50 show no drought in the area, while values between 0.35–0.50 correspond to a mild drought, between 0.20–0.35 to a moderate drought, between 0.10–0.20 to a severe drought, and less than 0.10 to an extreme drought (Iban 2022).

Many researchers have employed these three vegetation health indices to investigate agricultural productivity and observe how vegetation responds to weather conditions. Kogan et al. (2012) used VCI and TCI to forecast winter wheat, sorghum, and corn yields 3–4 steps before harvest in Kansas, USA, and they found a strong correlation between crop yield and vegetation health indices during crop development. Bokusheva et al. (2016) examined the ability of the VCI and the TCI to forecast wheat output in the main wheat-producing areas of Kazakhstan. These indices were used to create index-based insurance policies. The study, conducted on 47 wheat farms in Northern Kazakhstan, found that insurance policies based on these indices can significantly reduce risk for a group of farms. Pei et al. (2018) evaluated the effectiveness of the VCI and the VHI in identifying plant responses to weather-induced changes in China from 1982 to 2013. The research revealed that there was general plant stress throughout the country for an average of two steps annually, as shown by the VCI and VHI. Möllmann et al. (2019) studied a selection of farms and their respective counties in northeastern Germany and found that, on average, the VHI had the strongest correlation with winter wheat yield. Jiang et al. (2021) examined how vegetation growth varies in space and time using satellite data on the NDVI, LST, VCI, TCI, and VHI. They observed that the number of lands affected by drought decreased over time, while the number of lands with normal and favorable plant growth increased from 1982 to 2016. Kloos et al. (2021) investigated whether remote sensing-based drought indices (TCI, VCI, and VHI) can accurately identify agricultural drought and crop losses in Bavaria, Germany. They found that the TCI and VHI had a strong correlation with soil moisture and crop yield anomalies, indicating they have the ability to detect agricultural and vegetation-based drought. Similarly, Chere et al. (2022) found that the VHI can identify moderate to severe agricultural droughts, with 26.3% of the total crop-growing areas showing a decreasing VHI trend. Additionally, the correlation between the VHI and crop yields was found to be good in most of the northern, central, and southeastern regions of Ethiopia.

### Review on time series forecasting for vegetation health

Scholars have used time series data from the VHI to map the likelihood of droughts and analyze vegetation health trends (Karimi et al. 2022). These analyses typically involve using the VHI values throughout the growing season and using linear regression to identify the relationship between the VHI values and ground measurements (Tuvdendorj et al. 2019). Previous research has primarily focused on creating models to forecast time series of NDVI, LST, soil moisture, and nitrogen content values (Nevavuori et al. 2019; Schwalbert et al. 2020). However, these studies have not adequately addressed forecasting time series of VHI values.

The Autoregressive (AR) method is effective for stationary time series, but more advanced models such as Auto Regressive Moving Average (ARMA) and Auto-Regressive Integrated Moving Average (ARIMA) have been developed using flexible exponential smoothing, and they have been used for NDVI and LST time series (Fernández-Manso et al. 2011; Tian et al. 2016). These models, however, have the potential for overfitting and can be computationally expensive for long-term patterns and high-dimensional inputs (Jiang 2021; Li and Song 2023). Traditional Machine Learning (ML) models like Support Vector Regression (SVR), Ridge and LASSO regression, and Random Forest (RF) are more practical due to the availability of high-quality pre-built solutions in the data science community. These regression models have been used for vegetation cover studies using LST and NDVI (Ang et al. 2022; Sun et al. 2019). However, traditional ML-based models may not be able to handle the complex non-linear dependencies of large multi-variable datasets (Chakraborty et al. 2021). Modeling highly nonlinear phenomena with spatiotemporal variability is a difficult task due to the presence of disturbances, modeling errors, and various uncertainties in real-world systems. This is especially challenging when there are missing data values in the input dataset (Gavahi et al. 2021).

Recurrent Neural Network (RNN) based models have been widely used for time series forecasting (Ferchichi et al. 2022). RNNs are a deep learning technique that takes into account the sequential relationships between input data and their effect on the output data, making them suitable for sequence modeling tasks (Bengio et al. 1994). They have been implemented in vegetation cover research. For instance, Khaki et al. (2020) demonstrated the capability of a CNN-RNN framework to generalize the yield prediction, and Yu et al. (2022) found better prediction accuracy for vegetation indices once they used the RNNs. However, the RNNs may struggle to learn the interdependency between input and output data when the sequence gets longer. (Hochreiter 1998; Vidal and Kristjanpoller 2020).

To overcome this limitation, a specific type of RNN called Long Short-Term Memory (LSTM) was introduced by Hochreiter and Schmidhuber (1997). The LSTM allows information from a sequence to be carried over to consecutive sequences, enabling the model to learn the relationship between the sequential data and output data. Previous research has shown that LSTM models perform better than traditional regression-based methods. For example, Cui et al. (2020) stated that the assumptions of linearity and stationarity used in ARIMA models could not be applied to NDVI time series, and could not accurately forecast abnormal changes caused by disturbances. Schwalbert et al. (2020) compared the performance of multivariate linear regression, RF, and LSTM for forecasting soybean yields using NDVI, LST, and precipitation as independent variables. They found a superior performance of the LSTM model relative to other algorithms for all the forecast dates. Wang et al. (2022) showed that the accuracy of the yield estimation results of LSTM was generally better than those of conventional ML methods. Celik et al. (2022) investigated soil moisture forecasting based on satellite-derived data with LSTM and they obtained accurate soil moisture values for the next day. Previous research on weather-induced vegetation stress forecasting using LSTM involves utilizing precipitation-based drought indices, such as Standardized Precipitation Index (SPI), Standardized Precipitation Evapotranspiration Index (SPEI), or combining them with other hydro-meteorological factors such as temperature, humidity, and wind speed (Vo et al. 2023; Wu et al. 2022). Using only LSTM networks for forecasting time series data has certain limitations. LSTMs, while effective in capturing long-term dependencies in sequential data, are not specialized models for learning spatial patterns, as they primarily focus on temporal patterns. Therefore, the LSTMs are unsuitable for handling inherent patterns and structures in grid-like data, such as images or videos (Hu et al. 2020). This limitation becomes particularly evident in remote sensing datasets, where spatial dependencies are crucial for understanding the phenomena like vegetation health.

Convolutional Long Short-Term Memory (ConvLSTM) is a type of RNN that combines the LSTM architecture with the CNNs architecture. It is particularly useful when the input data has a grid-like structure. It uses convolutional filters to extract features from the input data and then processes them over time using the LSTM architecture. It can effectively learn spatial and temporal dependencies in the input data (Shi et al. 2015). In the previous research focusing on the NDVI or LST time series forecasting with ConvLSTM, the NDVI or LST values for each pixel in the region of interest were used as input, with the time dimension considered the third dimension. The model would take the NDVI or LST values as input and use convolutional filters to extract features. Then the LSTM architecture would be used to process the features over time and make forecasting for the NDVI or LST values in the future (Ahmad et al. 2020; Kartal and Sekertekin 2022). Gavahi et al. (2021) used the ConvLSTM network for soybean yield forecasting trained with LST and land cover data and improved the effectiveness and usefulness of ConvLSTM by combining it with 3D CNNs. Yinglan et al. (2022) generated a large spatiotemporal vertical soil moisture dataset for training and verifying a ConvLSTM model. NDVI and other remote sensing-based factors were used as predictive variables. Results from the ConvLSTM model showed that the accuracy of root-zone soil moisture estimation improved significantly compared to Global Land Data Assimilation System (GLDAS) products, particularly for deeper layers. Kartal and Sekertekin (2022) took advantage of the ConvLSTM model to predict 8-day LST images and to obtain better accuracies than the LSTM and multi-layer perceptrons.

**Fig. 1:**
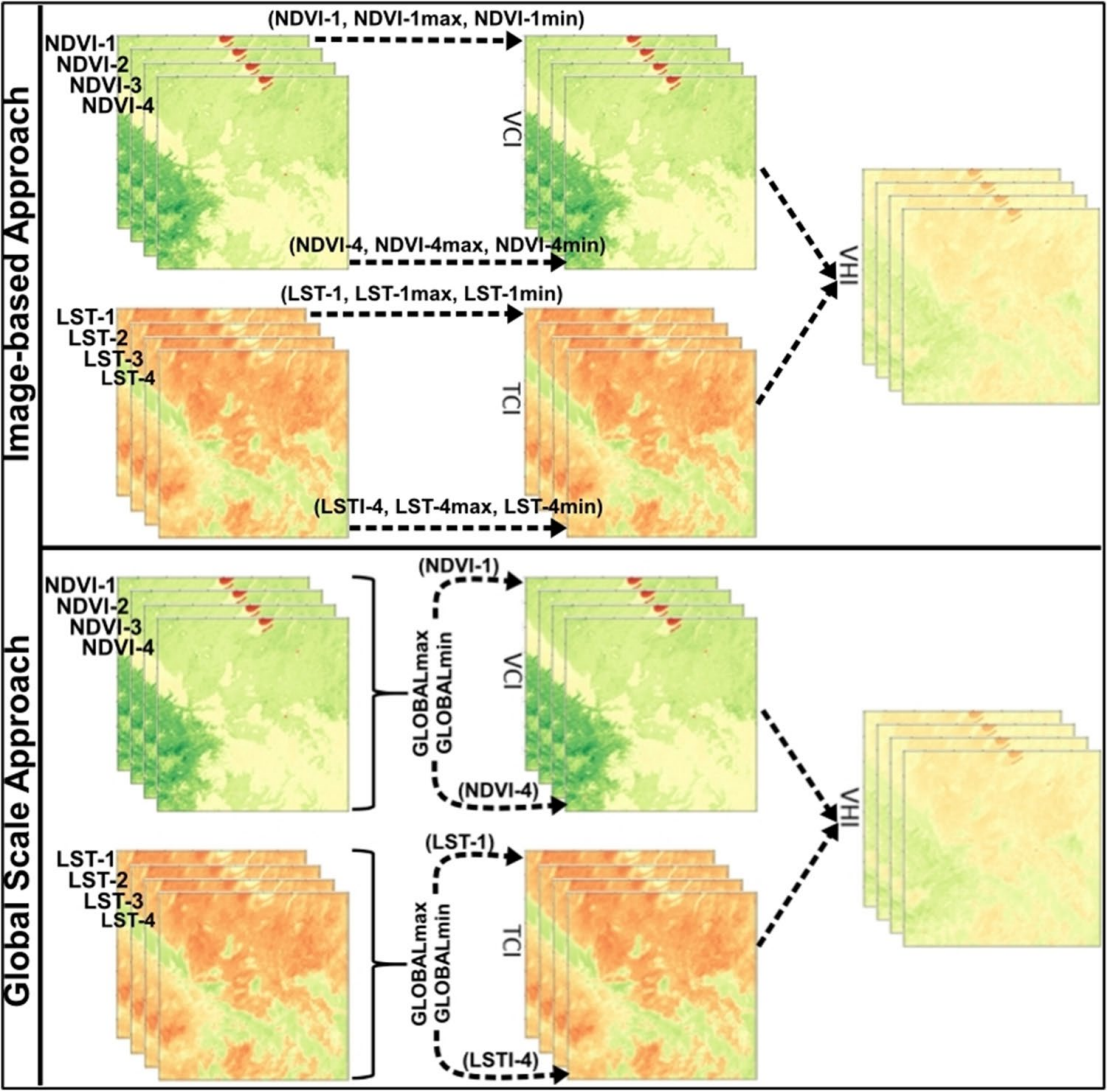
Illustration of the image-based approach and global scale approach. The parameters above the arrows are the inputs to calculate the VCI and TCI images using Eqs. (2) and (3), respectively

ConvLSTM utilizes convolutional operations to extract spatial pattern information from images, while convolutional gates enable the analysis of temporal information in a way similar to traditional LSTM. The advantage of ConvLSTM lies in its ability to achieve this with fewer parameters and increased computing power. Addressing the lack of time series forecasting for the VHI index, this study aims to be the first to forecast this index under various assumptions using remote sensing datasets and a ConvLSTM network. The methodology explores the performance of three ConvLSTM structures with one, two, and three layers. The forecasting intervals include 1 step, 2 steps, and 3 steps ahead, where each step corresponds to an 8-day forecasting horizon. These scenarios are tested using two approaches: iterative and separate forecasting. In the iterative approach, the last testing data is used to forecast the next step, whereas in the separate approach, only the training dataset is used to forecast the next step. Furthermore, two ways of utilizing the dataset are considered: grid or image-based and a novel approach called ‘global scale.’ Fig. 1 represents the illustration of the image-based approach and global scale approach. In traditional VHI calculation from the VCI and TCI images, minimum and maximum pixel values of NDVI and LST are obtained from the corresponding single image pairs. For example, for the retrieval of VCI from an NDVI image, minimum and maximum pixel values of this NDVI image are determined and then VCI is calculated based on these values, which we call grid or image-based approach (upper side of Fig. 1). However, in the global scale approach as seen in the lower part of Fig. 1, GLOBALmax and GLOBALmin values represent the maximum and minimum pixel values, respectively, which are extracted from the whole dataset, meaning all images.

## Study area and data

**Fig. 2:**
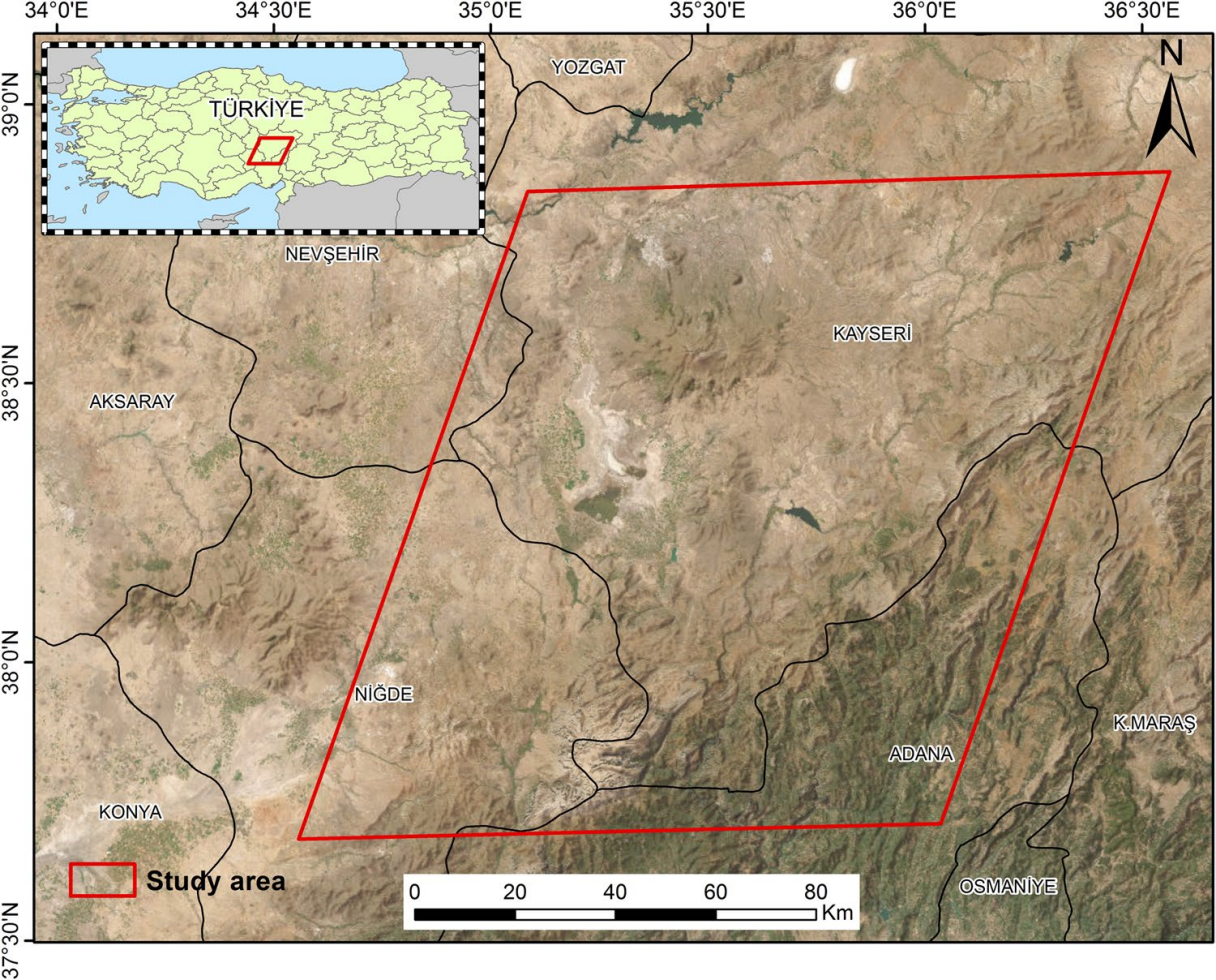
Study Area

The focus of our research lies in the southeastern part of Central Anatolia, Türkiye, encompassing the Cappadocia region and rural areas within the provinces of Niğde, Kayseri, Adana, and Nevşehir (Fig. 2). Central Anatolia has experienced devastating drought events throughout its history, which have even led to the collapse of ancient civilizations (Manning et al. 2023). The region is characterized by its elevated terrain, predominantly highlands and expansive plateaus, with an average altitude of 1150 m. Acting as a climatic barrier, the Taurus Mountains range in the south, significantly influencing the region's climate by impeding the influx of humid air masses from the nearby coastal regions. Central Anatolia exhibits a typical dry climate, with an average temperature of around 10 °C and an annual precipitation of approximately 400 mm, the lowest among other regions in the country. The region is dominated by convective and frontal rainfall systems, with the majority of precipitation occurring during the winter and spring seasons. In the coldest month, January, the mean temperature is 0.7 °C, while in the hottest month, July, it reaches 22 °C. The annual mean temperature is recorded as 10.8 °C (Bacanli et al. 2011; Yıldız 2014).

Given the historical prevalence of catastrophic drought events in Central Anatolia, it is crucial to investigate and predict prospective vegetation health conditions in that region. Understanding the underlying factors and patterns contributing to drought occurrence can aid in proactive drought management, resource allocation, and mitigation strategies. This research aims to provide a solid foundation for forecasting and preparing for drought events or unhealthy vegetation conditions in the study area, thus contributing to sustainable water resource management and the resilience of local communities and ecosystems.

The study utilized two satellite-derived datasets (MODIS—Moderate Resolution Imaging Spectroradiometer) covering ten years from January 1, 2012, to January 1, 2022. The datasets used were the MOD11A2.061 and MOD09GA_006 NDVI products, which provide valuable information on the LST and NDVI, respectively.

The MOD11A2.061 dataset, obtained from the MODIS Terra satellite instrument, offers global coverage of LST and emissivity at a spatial resolution of 1 km per pixel. This dataset consists of 8-day composites, resulting in a total of 460 images covering the 10 years. The LST values in this dataset represent the temperature of the land surface, while the emissivity values indicate the surface's ability to emit thermal radiation (Wan et al. 2015). The MOD09GA_006 NDVI dataset, also acquired from the MODIS Terra satellite instrument, provides information on vegetation dynamics using NDVI. This dataset offers daily measurements at a spatial resolution of 463.313 m (Jalayer et al. 2023). Over the 10-year duration, it yields a total of 3663 images.

To create a consistent LST and NDVI image dataset, the starting date of the LST data was assumed as the reference point. The NDVI data were then processed by calculating the 8-day averages to align with the temporal resolution of the LST data. This resulted in a final dataset of 460 paired LST and NDVI images.

The spatial resolution of the original LST data was 1000 m, while the NDVI data had a resolution of 463.313 m. To ensure consistency, the NDVI dataset was resampled using the *scale:1000* parameter in Google Earth Engine (GEE). This resampling aligns with the principles of image pyramids, where different resolution levels represent downsampled versions of the original image. By setting the scale to 1000 m, GEE internally selects the appropriate level from the image pyramid, ensuring a consistent and uniform resolution for the NDVI data. This resampling process facilitates coherent scientific analysis and interpretation. Then, Eqs. (2), (3), and (4) given in Section 2, were utilized to generate the VCI, TCI, and VHI values, respectively. In the end, the original image datasets were clipped to our designated study area, which had dimensions of 128 pixels in width and 128 pixels in height. This implies that the VHI images encompassed a study area of 128 square kilometers.

## Methodological framework

### Linear interpolation (LI)

Linear Interpolation (LI) is a common method used to fill in missing data in remote sensing datasets. This technique involves estimating the value of a missing data point by calculating the linear relationship between two known spatial data points within the given remote sensing image. For example, if we have satellite imagery with a few missing data points, we can use LI to estimate the values of these missing points based on the values of the surrounding data points. This can be done by fitting a straight line to the known spatial points and using this line to predict the value of the missing points. LI is a simple and efficient method, often used in conjunction with other techniques to improve the accuracy of the results. The formula of the LI method can be found in our previous study (Kartal and Sekertekin 2022).

### Convolutional Long Short-Term Memory (ConvLSTM)

ConvLSTM model is a type of deep learning model that combines the features of CNN and LSTM networks. The ConvLSTM is often used for tasks involving sequential data, such as time series and video forecasting. Although there are ConvLSTM structures developed in different ways in the literature, this study is based on the architecture developed by Shi et al (2015). It is composed of both convolutional and LSTM layers, which allows it to effectively capture both spatial and temporal dependencies in the data. The convolutional layers are responsible for extracting spatial features from the data, while the LSTM layers capture temporal dependencies by using their internal memory cells to remember past information.

A ConvLSTM cell consists of three main components: the input gate (${i}_{t}$), the forget gate (${f}_{t}$), and the output gate (${o}_{t}$). These gates are used to control the flow of information into and out of the cell, allowing the ConvLSTM cell to store and retrieve information over time. The formulas of gates and auxiliary functions are given below:

5 $${i}_{t}= \sigma \left({W}_{xi}* {x}_{t}+ {W}_{hi}* {h}_{t-1}+ {W}_{ci}* {c}_{t-1}+ {b}_{i}\right)$$

6 $${f}_{t}=\sigma \left({W}_{xf}*{x}_{t}+{W}_{hf}*{h}_{\left\{t-1\right\}}+{W}_{cf}*{C}_{\left\{t-1\right\}}+{b}_{f}\right)$$

7 $${o}_{t}= \sigma \left({W}_{xo}* {x}_{t}+ {W}_{ho}* {h}_{t-1}+ {W}_{co}* {c}_{t}+ {b}_{o}\right)$$

8 $${c}_{t}= {f}_{t}* {c}_{t-1}+ {i}_{t}*{\text{tanh}}\left({W}_{xc}* {x}_{t}+ {W}_{hc}* {h}_{t-1}+ {b}_{c}\right)$$

9 $${h}_{t}= {o}_{t}*{\text{tanh}}\left({c}_{t}\right)$$

where the convolution operation is represented by ‘*’, and the Hadamard product is represented by 'o'. Here ${x}_{t}$ denotes the input, ${h}_{t}$ denotes the output, and ${c}_{t}$ represents the cell state at time t. The input gate (${i}_{t}$), forget gate (${f}_{t}$), and output gate (${o}_{t}$) are responsible for managing information flow. The weights and biases of the ConvLSTM cell are denoted as *W* and *b*, respectively. The sigmoid activation function (*σ*) is used for gating mechanisms, while the hyperbolic tangent activation function (*tanh*) processes the final output.

The input gate utilizes a sigmoid activation function to determine which portions of the input should be stored in the cell's memory. Conversely, the forget gate, also employing a sigmoid activation function, decides which information in the cell's memory should be retained or discarded. The output gate, again using a sigmoid activation function, controls the information to be outputted. The output of the output gate undergoes a *tanh* activation function to yield the final output of the ConvLSTM cell.

In summary, the intricate structure of a ConvLSTM cell enables efficient storage and retrieval of information over time, making it particularly well-suited for tasks involving sequential data such as time series forecasting and video forecasting.

### Workflow of the forecasting methodology

**Fig. 3:**
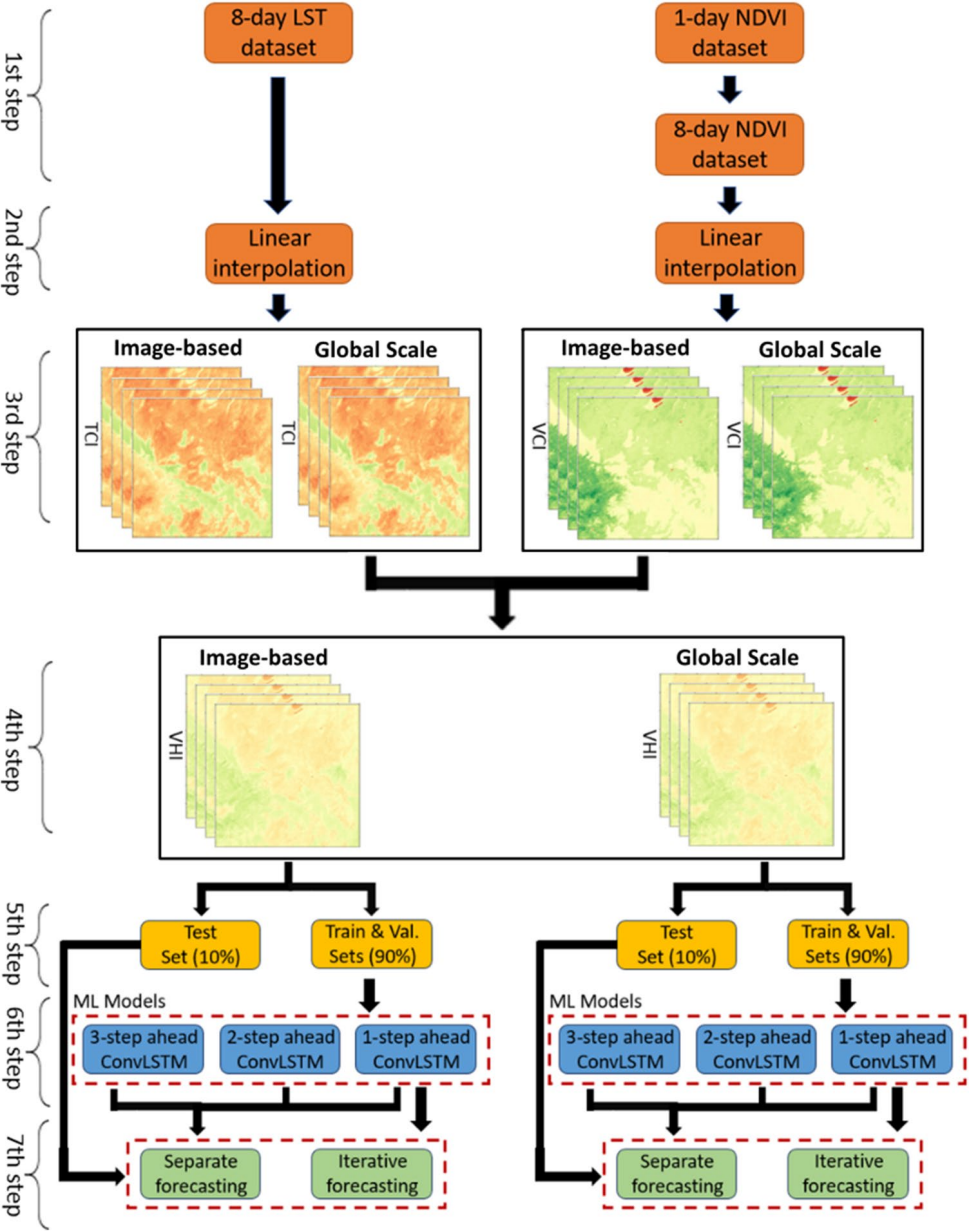
Workflow of the proposed methodology

The main steps of the study are given in Fig. 3. The 1st step is the pre-processing of the datasets. In this step, the raw NDVI data were converted into a suitable format for 8-day MODIS LST data, as described in “Study area and data” section. Then, LI was applied to complete the possible missing information in the datasets. In the 3rd step, the TCI and VCI values were calculated using the LST and NDVI data. In this step, two different dataset strategies were applied to calculate the TCI and VCI values. In the 1st dataset strategy, the required maximum and minimum values for Eqs. (2) and (3) were calculated from a single data (called grid or image-based). In the 2nd dataset strategy, these values were calculated from the entire dataset (called global scale). Consequently, two different datasets were obtained. The steps for the rest of the process are the same for both datasets. In the 4th step, TCI and VCI datasets were combined to create a single VHI dataset.

In the 5th step, three different datasets were created depending on the forecasting step size. Therefore, three different ConvLSTM models were obtained. Accordingly, the first model forecasts one step, whereas the second and third models forecast two steps and three steps ahead, respectively. The only difference in creating these models is that in the first model, the output values point to one-step ahead values, while in the second model, the output values point to two-step ahead values, etc. In other words, when the input values of 1, 2, 3, 4, 5, and 6 time steps are utilized, the first model is trained to forecast the 7th time step and the second model is trained to forecast the 8th time step.

The main idea in creating different models is to use two different forecasting-strategies (iterative and separate forecasting) to forecast 1-, 2-, and 3-time steps ahead. According to the first forecasting-strategy, firstly, the 7th time-step is forecasted using the data belonging to the 1–6 time steps. In order to forecast the 8th time step, the actual measurement values of the 2–6 time steps and the forecasted values of the 7th time step are used. Similarly, to forecast the 9th time step, the actual measurement values of the 3–6 time steps and the forecasting values obtained from the iterative forecasting strategy for the 7th and 8th time steps are used. In other words, since the model can forecast one-step ahead, the previous forecasting values are used to forecast further steps. This forecasting-strategy is called *iterative forecasting*.

In the second forecasting-strategy, to forecast the 8th time step, the model is trained to forecast two steps ahead directly using data from the actual measurement values of the 1–6 time steps. Similarly, to forecast the 9th time step, the model is trained to forecast three steps ahead. This forecasting strategy is called *separate forecasting*. Thus, forecasting processes were carried out in two different ways and the performance of the two strategies was evaluated in the results section.

As a result, in the 3rd step, two different datasets were created, and processed using two distinct forecasting strategies. In the time domain, while the first 80% of the dataset was used to train the models, the next 10% was used for validity, and the last 10% was used for testing. Three different model structures were used, consisting of layers 1, 2, and 3. The number of filters in the CNN part was set to 16 and the kernel size was set to 3. The model was trained for a maximum of 100 iterations and early stopping conditions were applied to prevent over-fitting. Accordingly, the performance of the model was evaluated according to the validation dataset after each epoch, and if there was no improvement in the performance of the model during 20 iterations, the training process was terminated.

### Evaluation metrics

Root Mean Squared Error (RMSE), Mean Absolute Error (MAE), and Mean Absolute Percentage Error (MAPE) metrics were used to evaluate the forecasting performance of the models. These metrics are commonly used to evaluate the performance of ML models, particularly in regression problems where the goal is to forecast a continuous value. RMSE is calculated by taking the square root of the average of the squared differences between the forecasted value and the actual value, as follows:

10 $$RMSE = \sqrt{\left(\frac{1}{n}\right)* \sum {\left({y}_{pred}- {y}_{true}\right)}^{2}}$$

where ${y}_{pred}$ is the forecasted value, ${y}_{true}$ is the actual value, and $n$ is the number of samples. The squared term in the RMSE loss function means larger errors are penalized more heavily than smaller ones. This can be useful in some cases but also make the RMSE sensitive to outliers.

MAE is defined as the average of the absolute differences between the forecasted value and the actual value, as follows:

11 $$MAE = \left(\frac{1}{n}\right)* \sum \left|{y}_{pred}- {y}_{true}\right|$$

The absolute term in the MAE loss function means that all errors, regardless of their size, are treated equally. This can make the MAE more robust to outliers, but it also means that the MAE may not be as sensitive to larger errors as the RMSE. On the other hand, MAPE incorporates the percentage difference between predicted and true values. The formula for MAPE is expressed as:

12 $$MAPE = \left(\frac{1}{n}\right)* \sum \left|\frac{{y}_{pred}- {y}_{true}}{{y}_{true}}\right|*100$$

By incorporating the percentage difference, MAPE provides a measure of error in percentage terms, offering insights into the relative accuracy of predictions. It is particularly useful when understanding the impact of errors relative to the actual values is important.

## Results

**Fig. 4:**
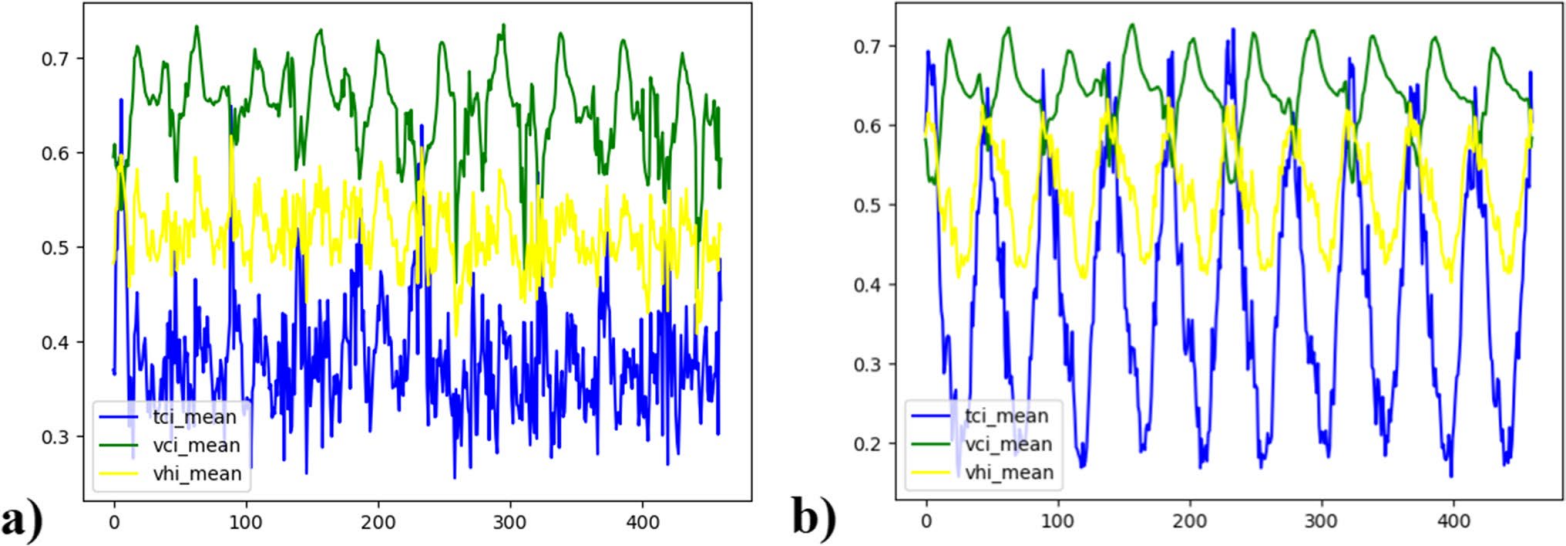
Distribution of the mean TCI, mean VCI, and mean VHI values from time series images: image-based min–max (**a**) and global min–max (**b**)

As mentioned above, in this study, two approaches, namely, the image-based approach and the global scale approach were investigated based on VHI forecasting. Within the global scale approach proposed in this study, we examined the effect of the global minimum and global maximum LST and NDVI values on the performance of the utilized ML or statistical method in VHI forecasting. The reason beyond this consideration is that utilization of the global minimum and global maximum values results in a more Gaussian distribution of the VHI time series, potentially leading to improved results with an ML method. Figure 4 presents the distribution of the mean TCI, mean VCI and mean VHI values obtained from the time series images in the abovementioned ways: image-based min–max (a) and global min–max (b).

Table 1 represents the average VHI forecasting results, calculated by averaging across all pixels in an image, based on the RMSE, MAE, and MAPE for the testing dataset. In the table, the accuracy results of iterative and separate forecasting approaches for image-based and global datasets were provided based on 1 step, 2 steps, and 3 steps ahead forecasting intervals, and three different layer structures. For both iterative and separate forecasting approaches, the global min–max dataset presented higher accuracies than the image-based dataset considering all forecasting intervals and all three layers. In Table 1, the RMSE values are increasing for both the global min–max dataset and image-based data set when the forecasting interval increases in both iterative and separate approaches, except for three-layered models with separate approaches. In addition, identical MAE results were obtained as in the RMSE results. For a more comprehensive evaluation, MAPE values were given alongside RMSE and MAE to quantify the relative forecasting accuracy in percentage terms. MAPE metrics also show that it is possible to make predictions with an approximate error rate of around 4% using the separate forecasting approach on the global scale dataset for up to 3 forecasting steps. The obtained results show that as the forecasting interval increases from 1 to 3 steps, the performance of the model decreases generally for both image-based and global datasets.

**Table 1:** The average VHI forecasting results, namely RMSE, MAE and MAPE, for the testing dataset

Statistical Metric	Forecasting Approach	Forecasting Interval (Step)	1 Layer		2 Layer		3 Layer	
			Image Based	Global Scale	Image Based	Global Scale	Image Based	Global Scale
RMSE	**ConvLSTM (Iteratively)**	**1**	0.045	0.025	0.054	0.038	0.061	0.049
		**2**	0.050	0.032	0.056	0.043	0.061	0.054
		**3**	0.053	0.037	0.061	0.049	0.063	0.058
	**ConvLSTM (Separately)**	**1**	0.045	0.025	0.054	0.038	0.061	0.049
		**2**	0.045	0.026	0.046	0.027	0.045	0.056
		**3**	0.046	0.028	0.067	0.026	0.045	0.030
MAE	**ConvLSTM (Iteratively)**	**1**	0.037	0.022	0.044	0.033	0.05	0.044
		**2**	0.042	0.028	0.046	0.038	0.051	0.048
		**3**	0.044	0.032	0.050	0.043	0.051	0.052
	**ConvLSTM (Separately)**	**1**	0.037	0.022	0.044	0.033	0.05	0.044
		**2**	0.037	0.022	0.037	0.022	0.037	0.027
		**3**	0.038	0.023	0.041	0.022	0.037	0.025
MAPE	**ConvLSTM (Iteratively)**	**1**	7.38	4.24	8.77	6.47	10.14	8.63
		**2**	8.35	5.46	9.27	7.47	10.24	9.53
		**3**	8.74	6.35	10.19	8.64	10.32	10.39
	**ConvLSTM (Separately)**	**1**	7.38	4.24	8.77	6.47	10.14	8.63
		**2**	7.42	4.35	7.46	4.34	7.38	5.11
		**3**	7.55	4.53	8.16	4.30	7.43	4.73

**Fig. 5:**
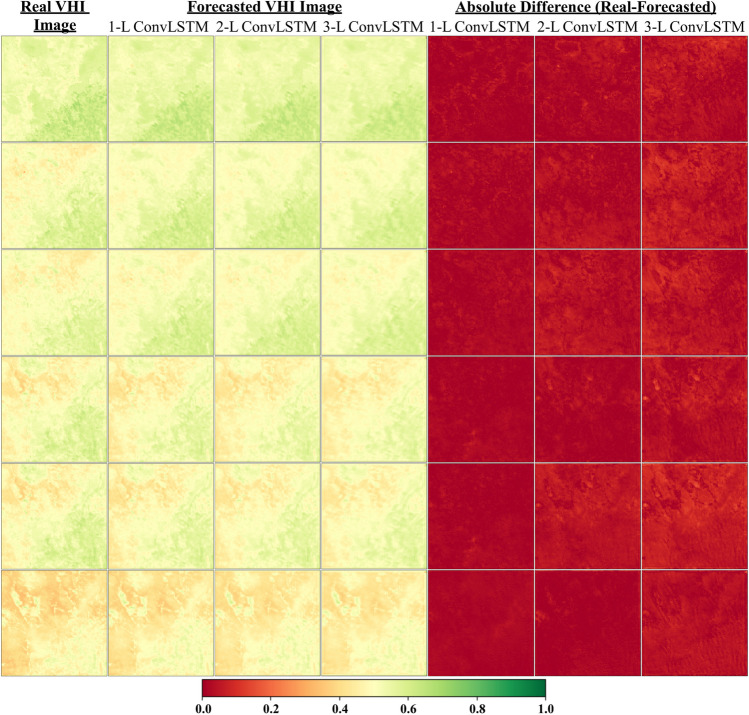
Comparison of real VHI and forecasted VHI images for 6 sample days of 1 step ahead. 1-L, 2-L, and 3-L refer to the 1-Layer Model, 2-Layer Model, and 3-Layer Model, respectively

**Fig. 6:**
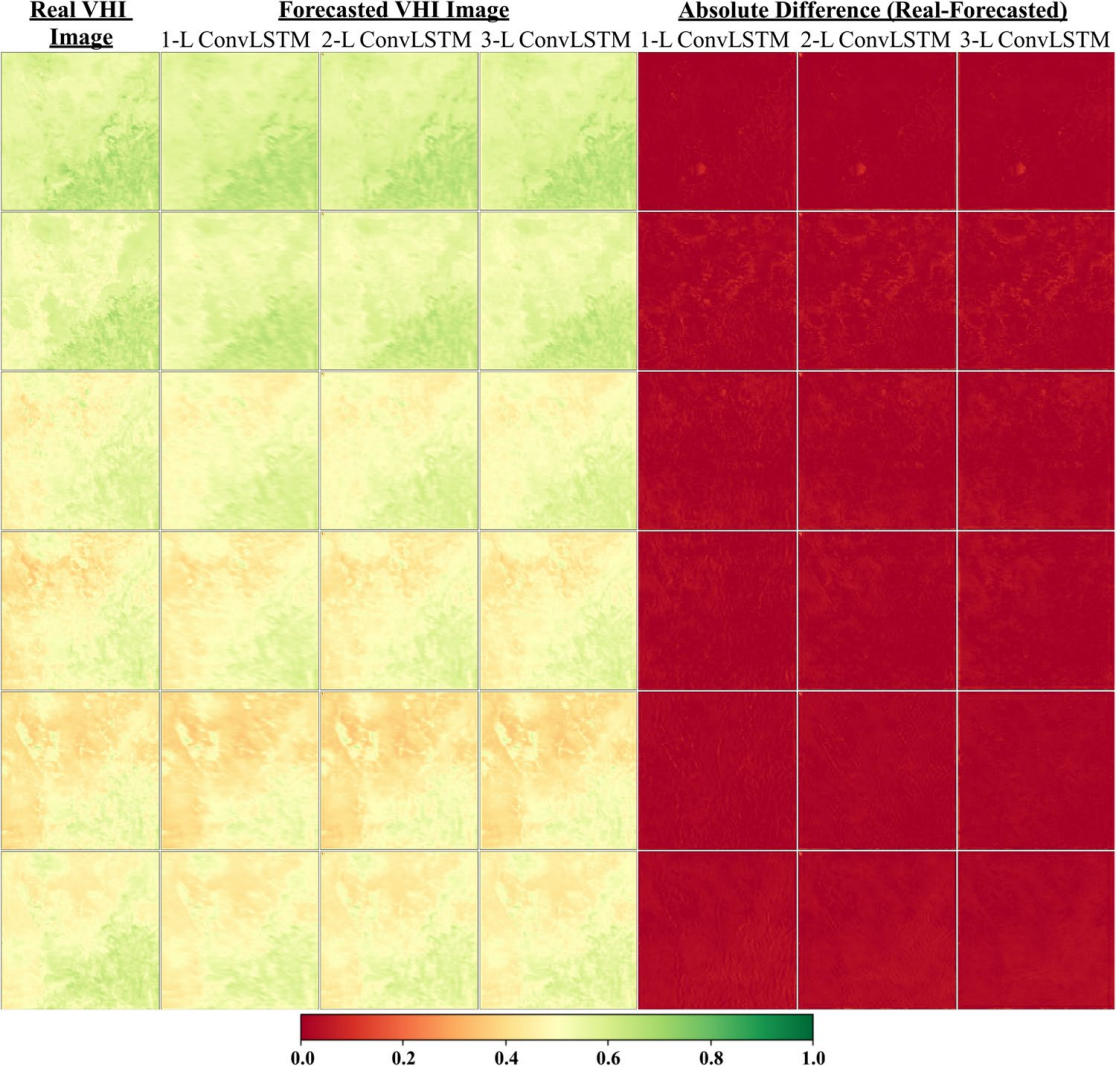
Comparison of real VHI and forecasted VHI images for 6 sample days of 2 steps ahead. 1-L, 2-L, and 3-L refer to the 1-Layer Model, 2-Layer Model, and 3-Layer Model, respectively

**Fig. 7:**
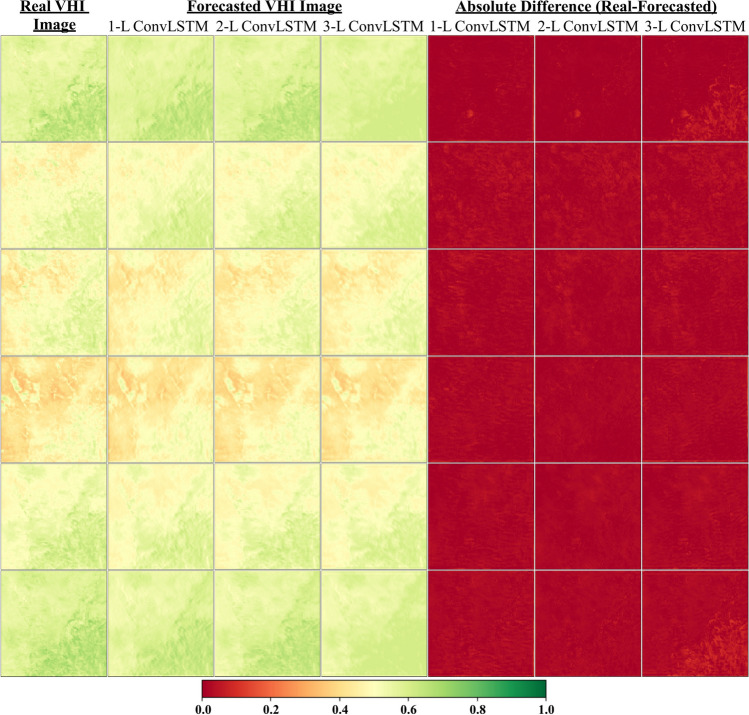
Comparison of real VHI and forecasted VHI images for 6 sample days of 3 steps ahead. 1-L, 2-L, and 3-L refer to the 1-Layer Model, 2-Layer Model, and 3-Layer Model, respectively

Table 1 highlights that the best results for all forecasting steps (from 1 to 3 steps) and both forecasting approaches (iterative and separate) are achieved with the global scale dataset and 1-layered structure. Considering one-step ahead (8-day later) forecasting with both iterative and separate approaches, the same RMSE value was obtained as 0.025 in the global scale dataset since the same processing steps were applied. However, concerning the forecasting of 2-step and 3-step ahead, the separate approach with the global dataset provided better RMSE values than the iterative one. In addition, the 2-layered model presented similar results to the 1-layered one for the separate approach in forecasting all steps ahead. To analyze the spatial variations, a comparison between the real VHI and forecasted VHI images on a global scale using the separate approach was demonstrated for six sample says. The results are presented in Figs. 5, 6, and Fig. 7 for forecasting intervals of 1-step, 2-step, and 3-step ahead, respectively. For the corresponding analysis, it is also clear from Table 1 that generally, the higher the layer number is, the lower the accuracy is. This case is also observed in spatial observations (Figs. 4, 5, and 6) when considering the absolute difference images.

**Fig. 8:**
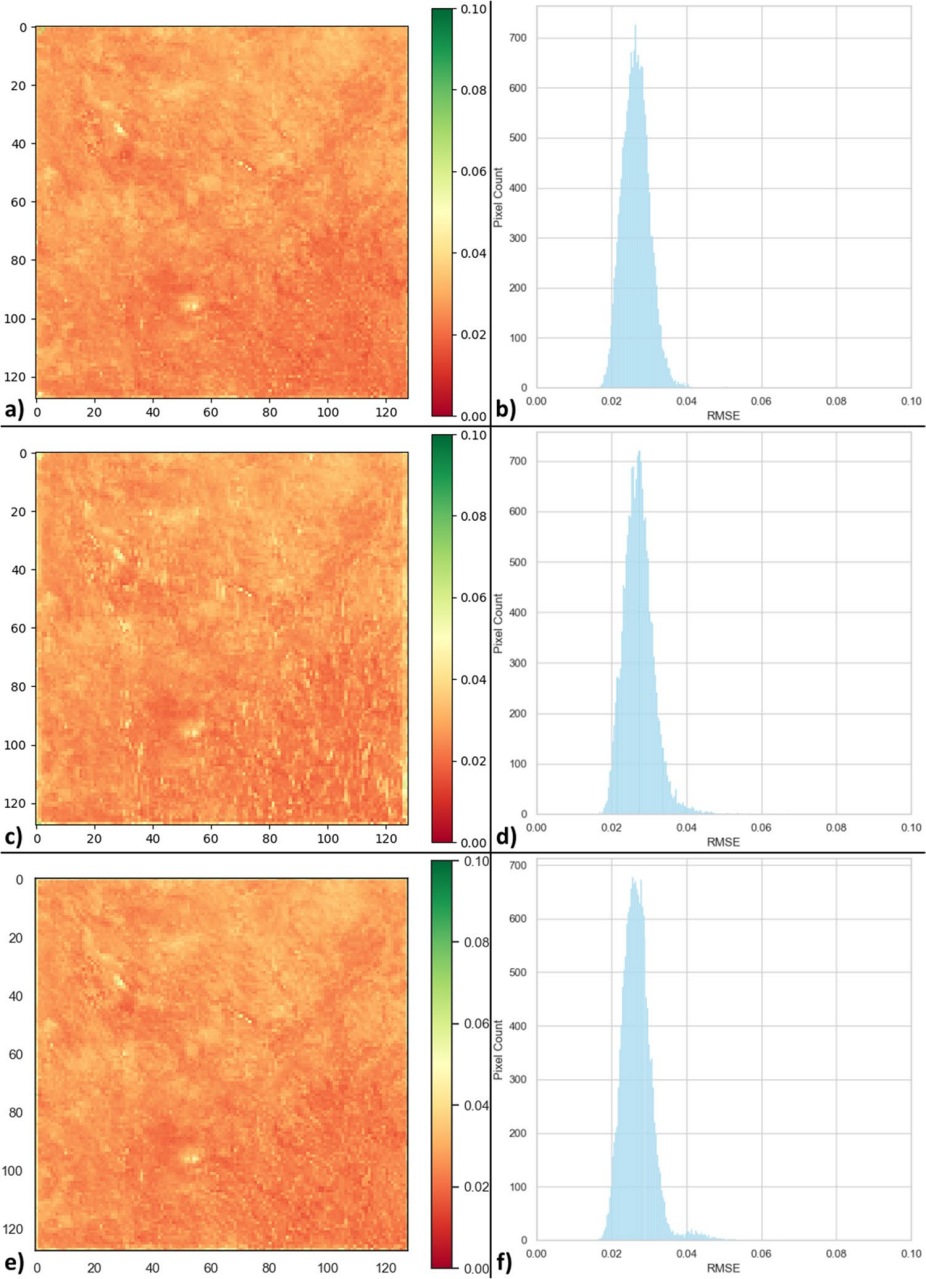
RMSE maps and their histograms of the best results for all forecasting steps (from 1 to 3 steps) calculated from the real VHI images and forecasted VHI images: (**a**-**b**) RMSE map and histogram of the best model for 1 step ahead forecasting, (**c**-**d**) RMSE map and histogram of the best model for 2-step ahead forecasting, (e–f) RMSE map and histogram of the best model for 3-step ahead forecasting

Concerning the spatial pattern of forecasting 1-step ahead in Fig. 5, almost all models provided satisfactory results; however, it is clear from the figure that the 1-layer (1-L) model achieved better results than the others did. Similar results are observed for forecasting 2-step ahead in Fig. 6. On the other hand, Fig. 7 shows that the 1-layer (1-L) and 2-layer (2-L) models yielded identical performances, both of which were superior to the 3-layer (3-L) model. All these performance observations can be recognized from the absolute difference images in the corresponding figures. To reveal the pixel-wise performance of the best models for all forecasting steps (from 1 to 3 steps) given in Table 1, RMSE maps and their histograms were generated for the testing set (Fig. 8). The best models for forecasting 1 step, 2 steps, and 3 steps ahead with separate approaches are the 1-L ConvLSTM model and 2-L ConvLSTM model, respectively. It is obvious from Figs. 8b, d, and f that the RMSE histograms for all forecasting steps vary from 0.02 to 0.04, displaying all best models present identical results. Besides, Figs. 8a, c, and e show that RMSE maps also present similar trends in pixel-wise spatial evaluation for all forecasting steps. These results prove the effectiveness of the proposed method.

Additionally, to assess the sustainability of consistent predictions, the study has been expanded to include extended predictions of 5, 10, and 20 steps ahead. The results are provided in Table 2. However, due to the resource and time requirements for repeating tests for all models, the necessary performance evaluation for 5, 10, and 20 steps ahead is provided only for the most successful configuration (global scale normalization—Separately—1-layer model). The findings reveal an escalation in error rates as the time step advances. Nonetheless, noteworthy success is retained even at the 10th step using the separate prediction method. For instance, while predicting the next step incurs a 4.24% error rate, the 10-step ahead prediction maintains a 5.32% error rate. However, as the step count reaches 20, the error rate is observed to rise to 11%.

**Table 2:** The average VHI forecasting performance of the best model for further prediction Steps (5, 10, 20)

	Forecasting Interval (Step)	MSE	MAE	MAPE
Separately	1	0.025	0.022	4.24
	2	0.026	0.022	4.35
	3	0.028	0.023	4.53
	5	0.029	0.023	4.59
	10	0.032	0.027	5.32
	20	0.065	0.060	11.13

## Discussion

In this study, various assumptions were considered to forecast remotely sensed VHI images. Concerning the methodology, the performance of three ConvLSTM structures with one layer, two layers, and three layers were examined. Considering the forecasting intervals, the ConvLSTM structures were performed to forecast 1 step, 2 steps, and 3 steps ahead with two approaches, namely iterative and separate. Regarding the data usage, we utilized grid or pixel-based and global scale to be applied in the ConvLSTM structures. Under these assumptions, this study is the first investigation in the literature that forecasts remotely sensed time series VHI data with a ConvLSTM network. In this study, we also proposed using the global scale when working with time series VHI images, and compared the results of that approach with the traditional image-based VHI images. In the global scale, global minimum and global maximum values in the time series dataset were utilized apart from the traditional image-based approach. As a result, the outcomes derived from the global scale outperformed the image-based results, providing evidence for the effectiveness of our approach.

Drought monitoring and forecasting are essential for the management and sustainability of the ecosystem, agriculture, water resources, and so on. Remote sensing datasets offer a wide variety of advantages for drought monitoring, planning, early warning, and forecasting. Time series analysis of remotely sensed datasets, which are among the drought indicators, is therefore crucial to determine the spatial and temporal intensity of drought on various scales. Some studies utilized Landsat-based VHI for drought monitoring (Ghaleb et al. 2015; Ejaz et al. 2023; Ayad et al. 2023). However, in this study, TCI, VCI, and VHI were obtained from the MODIS data, whose temporal resolution is better than the Landsat satellites. On the other hand, the spatial resolution of Landsat data is better than MODIS, which makes it possible to select the dataset based on the context of the investigation. Nevertheless, the higher temporal resolution increases the number of data for a specific period, which enables ML methods to learn the spatiotemporal pattern effectively.

The VHI has been considered one of the substantial drought indicators in previous studies; however, they were generally related to drought-based monitoring, detection, modeling, mapping, and risk assessment (Aksoy et al. 2019; Aitekeyeva et al. 2020; Kocaaslan et al. 2021; Rojas 2021; Chere et al. 2022; Fathi-Taperasht et al. 2023). There is a lack of research on using VHI to forecast future drought conditions using machine learning techniques and time series data. Some of the abovementioned studies also utilized time series VHI images; nevertheless, none of them implemented combined CNN and RNN (LSTM) structures for time series forecasting of the VHI as performed in this study.

## Conclusions

This study represents the first-ever attempt to forecast remotely sensed VHI time series images using the ConvLSTM network and a novel approach to time series data called "global scale". The VHI is a composite index that combines the VCI and TCI to encompass vegetation conditions and temperature stress. The VCI and TCI were derived from daily MODIS NDVI and 8-day MODIS LST datasets, respectively. To ensure consistency in the application, daily MODIS NDVI images were averaged to create the 8-day dataset, and any missing data in these images were filled using linear interpolation. The study considered several assumptions, as mentioned in the Discussion, for forecasting the VHI over three time intervals: 1 step, 2 steps, and 3 steps ahead. In the application, two different approaches, namely separate and iterative, were employed to forecast new VHI images for the subsequent steps. The separate approach yielded superior results compared to the iterative approach. Furthermore, the study introduced the global scale dataset along with the separate approach, leading to the most exceptional outcomes when combined with the ConvLSTM network. The VHI forecasting based on the corresponding time intervals exhibited satisfactory results, as indicated by the average statistical metrics and RMSE maps.

## Data Availability

Data can be shared upon reasonable request.
